# Checking Pulmonary Hemorrhage (Extract)

**Published:** 1882-12

**Authors:** 


### CHECKING OF PULMONARY HEMORRHAGE.

Dr. A. C. Post writes: "In a recent number of *The Record* is an article entitled 'A Simple Means of Checking Pulmonary Hemorrhage with Shawl-straps.' For more than thirty years I have been in the habit of arresting internal hemorrhages by bandaging the arms and thighs so as to shut off from the general circulation a very considerable portion of blood in the veins of the extremities."

